# Examining associations between MDMA/ecstasy and classic psychedelic use and impairments in social functioning in a U.S. adult sample

**DOI:** 10.1038/s41598-023-29763-x

**Published:** 2023-02-11

**Authors:** Grant Jones, Joshua Lipson, Erica Wang

**Affiliations:** 1 Department of Psychology, Harvard University, 33 Kirkland St, Cambridge, MA 02138 USA; 2 Teachers College, Columbia University, New York, USA; 3 Colgate University, New York, USA

**Keywords:** Psychology, Human behaviour

## Abstract

Impairment in social functioning is a common source of morbidity across many mental health disorders, yet there is a dearth of effective and easily implemented interventions to support social functioning. MDMA/ecstasy and classic psychedelics (psilocybin, LSD, peyote, mescaline) represent two potential treatments for impairments in social functioning, as evidence suggests these compounds may be supportive for alleviating social difficulties. Using a nationally representative sample of U.S. adults from the National Survey on Drug Use and Health (2015–2019) (*N* = 214,505)*,* we used survey-weighted multivariable ordinal and logistic regression to examine the associations between lifetime use of the aforementioned compounds and impairments in social functioning in the past year. Lifetime MDMA/ecstasy use was associated with lowered odds of three of our four social impairment outcomes: difficulty dealing with strangers (aOR 0.92), difficulty participating in social activities (aOR 0.90), and being prevented from participating in social activities (aOR 0.84). Lifetime mescaline use was also associated with lowered odds of difficulty dealing with strangers (aOR 0.85). All other substances either shared no relationship with impairments in social functioning or conferred increased odds of our outcomes. Future experimental studies can assess whether these relationships are causal.

## Introduction

Impairments in social functioning are a hallmark feature of a wide range of mental health disorders, such as generalized anxiety, major depressive disorder, and schizophrenia^1–5^. Indeed, social impairment represents a large share of the cost to society of these disorders, as measured by the burden accrued by patients, caretakers, and treatment institutions^6,7^. However, options for treatment of impairments to social functioning have critical limitations to their efficacy^8–11^. Increasingly, the need to explore alternative approaches to supporting social functioning has been recognized, with promising results in the realm of novel pharmacotherapies^12–15^. In particular, MDMA (“ecstasy”) and classic psychedelics represent two potential avenues for further exploration of potential treatments for impairments in social functioning, as preliminary evidence has demonstrated that these compounds might help to alleviate social difficulties. To this end, the present study aims to explore possible protective associations between MDMA/ecstasy and classic psychedelic use and impairments in social functioning in a naturalistic context.

First synthesized by Merck in 1912, MDMA/ecstasy is a synthetic amphetamine compound which has been linked to feelings of euphoria and connection, disinhibition, and fear extinction^16,17^. At the neurotransmitter level, MDMA/ecstasy acts by binding to serotonin transporters, and to a lesser degree dopamine and norepinephrine transporters, promoting the release of these neurotransmitters and blocking their removal^18,19^. Most prominently, in the last few years, an impressive body of clinical work has demonstrated the efficacy of MDMA-assisted psychotherapy in treating symptoms of PTSD^20–24^.

Concurrently, a broader literature has explored the relationship between MDMA/ecstasy and prosociality across a range of domains and methods. A meta-analysis conducted by Regan et al., aggregating data across 27 experimental studies and 592 participants, found a moderate-to-large effect (*d* = 0.86) of MDMA/ecstasy on self-reported sociability-related measures, such as feeling loving, talkative, and friendly^25^. Meanwhile, Britt and Bedi summarize a wide-ranging set of findings demonstrating MDMA/ecstasy’s effects on social behavior in humans and laboratory animals, including increased cognitive empathy (the ability to understand the feelings of another and take another’s perspective), reduced aggression, and blunted emotional responses to social rejection^26^. In addition, MDMA/ecstasy administration has also been linked to short-term improvements in emotional empathy (the ability to feel what another person feels) and increased social approach behavior (i.e., willingness to facilitate social interaction)^27,28^.

Drawing upon this evidence, researchers have begun to explore the clinical application of MDMA/ecstasy to the treatment of impairments in social functioning. In light of the link between MDMA/ecstasy and increased prosocial behavior, a prospective review paper by Heifets and Malenka (2016) proposes that MDMA/ecstasy may alleviate impairments in social functioning in the context of a wide range of mental health disorders (e.g., autism, schizophrenia, social anxiety disorder, and major depressive disorder)^29^. Additionally, a groundbreaking pilot study demonstrated a large (*d* = 1.4) effect of two MDMA-assisted psychotherapy sessions on social anxiety symptoms in adults with autism^30^, laying the groundwork for larger-scale studies and applications to social difficulties beyond the context of this disorder.

Classic psychedelics (Greek for “mind-manifesting”), meanwhile, are a set of serotonergic compounds either found in nature or synthesized from natural compounds, which lead to profound alterations in perception and may induce mystical-type experiences of lasting personal and spiritual significance^31,32^. Alongside their exploration in the treatment of addiction^33,34^, depression^35,36^, and end-of-life anxiety^37^, classic psychedelics have also been probed for their applications in the realm of social functioning. Researchers have linked the mystical experiences occasioned by classic psychedelics to increases in measures of prosocial attitudes and behaviors, while others have linked classic psychedelics to enhanced emotional empathy and sociality^38,39^. In addition, a pharmacological fMRI study by Preller et al. found that classic psychedelics modulate multiple aspects of social cognition via serotonin 2A receptor agonist activity, and these researchers thus suggest that these substances may treat social impairments related to mental health disorders^40^. Further basic and clinical evidence supports the possibility that classic psychedelics might have a role to play in the treatment of social impairments related to mental illness as well^41,42^.

In summary, an emerging body of evidence suggests that MDMA/ecstasy, and perhaps also classic psychedelics, have a role to play in treating impairments in social functioning. However, the nature of this evidence remains preliminary, especially in light of the limited number of clinical investigations in this research area as well as the small sample sizes of prior work.

Therefore, the current study aims to investigate the associations between use of MDMA/ecstasy and classic psychedelics and four different facets of social impairment, using a large-scale nationally representative sample. While this method cannot be used to infer direct causal associations between use of MDMA/ecstasy and classic psychedelics and quality of social functioning, analyses based on a large (*n* > 200,000) and representative population sample can complement basic science and small-sample clinical findings within this research area and facilitate future experimental studies within this research domain.

## Methods

Data for this project are from the National Survey on Drug Use and Health (2015–2019), an annual survey that assesses substance use and mental health in a nationally representative sample of the United States population aged 12 and older^43–47^. The NSDUH survey is administered by interviewers in participants’ homes using a computer-assisted interviewing paradigm. Currently incarcerated individuals, active duty military members, and individuals experiencing homelessness that are not residing in a shelter are not surveyed by the NSDUH. We included all adults 18 years and older in our analyses (unweighted N = 214,505). This study was exempt from IRB review as all NSDUH data are publicly available at the following web address: https://www.datafiles.samhsa.gov. Furthermore, this study was conducted in accordance with all of the relevant guidelines and procedures.

### Dependent variables

We included all four dependent variables from the NSDUH that assessed impairments in social functioning caused by mental health problems or emotional difficulties. All participants were asked to think of the one month in the past 12 when they were “the most depressed, anxious, or emotionally stressed” when answering questions related to their social functioning. The four dependent variables are as follows:

1. The degree of difficulty interacting with strangers (“how much difficulty did you have dealing with people you did not know well?”) (ordinal: 1–4)
2. Being prevented from interacting with strangers due to mental health issues (“Did problems with your emotions, nerves, or mental health keep you from dealing with people you did not know well?”) (binary—yes/no)
3. The degree of difficulty participating in social activities (“how much difficulty did you have participating in social activities, like visiting friends or going to parties?”) (ordinal: 1–4)
4. Being prevented from engaging in social activities due to mental health issues (“Did problems with your emotions, nerves, or mental health keep you from participating in social activities?”) (binary—yes/no)

The variables assessing difficulty were assessed on a scale of “1” (no difficulty) to “4” (severe difficulty); although participants could also respond with a score of “5” (did not engage with strangers/participate in social activities), we re-coded these responses as N/As, as these responses do not capture whether the lack of engagement or participation was caused by emotional difficulties or mental health problems.

### Independent variables/covariates

The primary independent variable for our analyses was lifetime use of MDMA/ecstasy (yes/no). Additionally, lifetime use of four commonly used classic psychedelics (psilocybin, LSD, peyote, mescaline) served as exploratory independent variables in our analyses. We included the following demographic factors and substance use variables as covariates: marital status, educational attainment, sex, age, income level, race/ethnicity, self-reported engagement in risky behavior, and lifetime use of various legal and illegal substances (PCP [phencyclidine], cocaine, inhalants, tranquilizers, heroin, pain relievers, stimulants, sedatives, and marijuana).

These covariates have been used in various population-based studies on psychedelics^48–53^, allowing for comparability between the results of this study and prior research within this domain. Additionally, the demographic covariates control for many common confounds (e.g., race, socioeconomic status), while the lifetime use variables control for the impact that co-occurring substance use may have on our results. Overall, these covariates reduce the likelihood that any associations between our independent and dependent variables are spurious.

### Analyses

For our project, we used the Survey package in R version 4.1.2^54^, which allowed us to incorporate the survey weighting and complex design of the NSDUH into our analyses. We also used the gtsummary package in R to generate all descriptive tables and statistics^55^.

To report demographic information on our sample of interest, we calculated the weighted percentage of each category of our main demographic factors, stratified by those who have versus have not used MDMA/ecstasy. Furthermore, we also used chi-squared analyses to compare whether there were significant demographic differences between individuals who have versus have not used MDMA/ecstasy.

We used multivariable ordinal and logistic regression to assess the relationships between MDMA/ecstasy use, classic psychedelic use, and our dependent variables assessing impairments in social functioning. We used ordinal regression for our two models that include the ordinal dependent variables assessing the difficulty of engaging with strangers/participating in social activities. Our large sample size provided adequate power for the models included within our study, allowing us to satisfy this fundamental criterion for conducting regression models. ~ 0.5% of total responses were omitted from the models due to missing data. Additionally, we used multivariable logistic regression for our two models that include the binary dependent variables assessing whether one was prevented from interacting with strangers/engaging in social activities due to mental health issues. All independent variables and covariates were entered simultaneously into our models.

## Results

The demographics of our sample, stratified by those who have versus have not used MDMA/ecstasy are presented in Table 1. Individuals who have used MDMA/ecstasy are more likely to fall into the following demographic categories: never married, more formally educated, younger, male, Non-Hispanic White, and more likely to engage in risky behavior.

**Table 1:** Demographics of those who have versus have not used MDMA/ecstasy.

Characteristic	Has not used MDMA/ecstasy (unweighted N + [weighted %]) (Total N = 193,310)	Has used MDMA/ecstasy (unweighted N + [weighted %]) (N = 21,195)	Chi-square	ndf^1^	ddf^2^	p-value^3^
Marital status			832	2.5	125	< 0.001
Married	82,307 (53%)	5773 (33%)				
Widowed	6500 (6%)	197 (1%)				
Divorced or separated	20,872 (14%)	2260 (13%)				
Never been married	83,631 (27%)	12,965 (52%)				
Education			157	2.0	99	< 0.001
Less than HS	5898 (4%)	220 (1%)				
Some HS/HS grad	71,777 (34%)	7018 (29%)				
Some College or Above	115,635 (62%)	13,957 (70%)				
Age			1428	2.5	127	< 0.001
18–25	62,044 (13%)	7872 (21%)				
26–34	37,264 (14%)	6752 (34%)				
35–49	50,849 (24%)	5717 (33%)				
50 +	43,153 (48%)	854 (12%)				
Sex			312	1.0	50	< 0.001
Male	88,453 (48%)	11,316 (57%)				
Female	104,857 (52%)	9879 (43%)				
Race/Ethnicity			108	4.9	244	< 0.001
Non-Hispanic White	114,517 (63%)	14,407 (72%)				
Non-Hispanic Black	25,385 (12%)	1696 (7%)				
Non-Hispanic Native American/Alaska Native	2774 (1%)	301 (1%)				
Non-Hispanic Native Hawaiian/Pacific Islander	999 (0%)	95 (0%)				
Non-Hispanic Asian	9680 (6%)	595 (3%)				
Non-Hispanic more than one race	5952 (2%)	1089 (3%)				
Hispanic	34,003 (16%)	3012 (14%)				
Yearly Household Income			1.1	2.8	140	0.3
< $20,000	38,606 (16%)	4328 (16%)				
$20,000-$49,999	60,139 (29%)	6807 (29%)				
$50,000-$74,999	30,244 (16%)	3271 (16%)				
$75,000+	64,321 (38%)	6789 (39%)				
Self-reported Engagement in Risky Behavior			1812	2.8	141	< 0.001
Never	100,795 (58%)	5116 (26%)				
Seldom	63,802 (31%)	8966 (44%)				
Sometimes	24,671 (10%)	6054 (27%)				
Always	3141 (1%)	1023 (4%)				

^1^ndf = numerator degrees of freedom.

^2^ddf = denominator degrees of freedom.

^3^Chi-squared test with Rao & Scott's second-order correction.

The results of our four models assessing the relationships between MDMA/ecstasy and classic psychedelic use and impairments in social functioning are presented in Table 2. Overall, MDMA/ecstasy conferred lowered odds of three of our four outcomes: difficulty dealing with strangers (aOR: 0.92; 95% CI [0.87, 0.97]), difficulty participating in social activities (aOR: 0.90 [0.85, 0.95]), and being prevented from engaging in social activities due to mental health issues (aOR: 0.84 [0.71, 0.99]). MDMA/ecstasy was not associated with being prevented from interacting with strangers. Mescaline use was also associated with lowered odds of difficulty dealing with strangers (aOR: 0.85 [0.76, 0.95]). All other substances, including classic psychedelics, either did not share a relationship to social impairment or conferred increased odds of social impairment. Lifetime LSD use was associated with increased odds of two social impairment outcomes: difficulty dealing with strangers (aOR: 1.13 [1.06, 1.20]) and difficulty participating in social activities (aOR: 1.11 [1.05, 1.17]).

**Table 2:** Results from multivariable ordinal and logistic regression models testing the associations between MDMA/ecstasy, classic psychedelics (psilocybin, LSD, peyote, mescaline), and impairments in social functioning.

	Difficulty dealing with strangers	Prevented from dealing with strangers	Difficulty participating in social activities	Prevented from social activities
Lifetime Use	aOR (95% CI)^1^	aOR (95% CI)	aOR (95% CI)	aOR (95% CI)
MDMA/Ecstasy	0.92** (0.87, 0.97)	0.93 (0.75, 1.16)	0.90*** (0.85, 0.95)	0.84* (0.71, 0.99)
Psilocybin	0.98 (0.92, 1.03)	0.99 (0.81, 1.21)	0.97 (0.92, 1.03)	0.99 (0.83, 1.18)
LSD	1.13*** (1.06, 1.20)	1.01 (0.81, 1.27)	1.11*** (1.05, 1.17)	1.04 (0.87, 1.23)
Peyote	1.01 (0.93, 1.11)	0.85 (0.53, 1.38)	0.96 (0.88, 1.06)	0.97 (0.72, 1.30)
Mescaline	0.85** (0.76, 0.95)	0.97 (0.61, 1.53)	0.95 (0.85, 1.05)	1.04 (0.81, 1.33)
PCP	1.07 (0.95, 1.21)	1.59** (1.16, 2.17)	1.07 (0.96, 1.19)	1.33 (0.99, 1.77)
Cocaine	1.02 (0.97, 1.07)	1.09 (0.88, 1.35)	1.05 (1.00, 1.10)	1.05 (0.91, 1.22)
Inhalants	1.37*** (1.31, 1.44)	1.06 (0.87, 1.29)	1.43*** (1.36, 1.50)	1.23** (1.06, 1.42)
Tranquilizers	1.89*** (1.82, 1.97)	2.27*** (2.02, 2.55)	1.99*** (1.91, 2.07)	2.54*** (2.33, 2.76)
Heroin	1.29*** (1.17, 1.42)	1.08 (0.82, 1.40)	1.19** (1.06, 1.34)	1.02 (0.85, 1.23)
Pain Relievers	1.34*** (1.29, 1.39)	1.26** (1.07, 1.49)	1.43*** (1.38, 1.49)	1.32*** (1.16, 1.50)
Stimulants	1.31*** (1.26, 1.35)	1.16 (0.99, 1.37)	1.31*** (1.26, 1.35)	1.45*** (1.29, 1.62)
Sedatives	1.58*** (1.51, 1.64)	1.97*** (1.73, 2.23)	1.61*** (1.55, 1.69)	1.74*** (1.55, 1.96)
Marijuana	1.39*** (1.35, 1.43)	1.22** (1.06, 1.41)	1.38*** (1.34, 1.43)	1.24** (1.10, 1.39)

All aforementioned demographic factors are included as covariates in these models; results for all substance use covariates are displayed below.

Values highlighted in bold indicate that the independent variable confers significantly lowered odds of the dependent variable.

*aOR* adjusted odds ratio, *CI* confidence interval.

^1^*p < 0.05; **p < 0.01; ***p < 0.001.

## Discussion

The goal of this study was to assess the relationship between use of MDMA/ecstasy and classic psychedelics, on one hand, and impairments in social functioning, on the other. Overall, lifetime use of MDMA/ecstasy conferred lowered odds of three of four outcomes related to impairments in social functioning. Mescaline was also associated with lowered odds of one outcome. All other substances either did not demonstrate an association with impairments in social functioning or conferred increased odds of social impairment. Furthermore, this study represents one of many that demonstrates lifetime psychedelic use to be associated with lowered odds of deleterious outcomes in a population-based survey sample^48,50–53,56,57^.

### Potential explanations: MDMA/ecstasy and lowered odds of social impairment

There are a few possible explanations for our results linking MDMA/ecstasy use to lowered odds of social impairment. We first summarize third variable factors which might explain this pattern of association; next, we follow this summary with a discussion of potential neurotransmitter-receptor-level mechanisms, and conclude by reviewing potentially relevant neural and behavioral factors downstream of them.

#### Third variable factors

Third variable factors, such as personality traits, political affiliations, and spirituality may drive the observed associations between MDMA/ecstasy and lowered odds of social impairment. ter Bogt et al. (2006) demonstrated that there were personality differences between MDMA/ecstasy users and non-users in a house party setting. Namely, the study revealed higher rates of extraversion associated with MDMA/ecstasy users^58^, a third-variable trait that may be linked to lowered odds of social impairment. Another study by Nour et al. (2017) discovered an association between psychedelic use and liberal political views^59^, providing another example of possible pre-drug differences that may exist in our sample. Altogether, existing studies suggest that third-variable, pre-drug traits may possibly account for some share of the observed correlation between MDMA/ecstasy use and reduced odds of social impairment in this study.

#### Neurotransmitter-receptor-level effects

The association between use of MDMA/ecstasy and reduced odds of social impairment is possibly linked to the drug’s effects on several critical neurotransmitters in the brain, namely, dopamine and serotonin—which lie upstream of other potential mechanisms at the neural and behavioral levels, mentioned later. Some evidence exists to suggest that MDMA-induced changes to these neurotransmitter-receptor systems in the brain are indeed long-lasting^60^, offering a plausible explanation for how limited intake of MDMA could be linked to persistent changes in social behavior.

Given that MDMA mainly impacts serotonin levels, it is worth considering that the association between lifetime use of MDMA and lowered odds of social impairment can be ultimately linked to changes in serotonergic neurotransmission. Lower levels of circulating serotonin in the prefrontal cortex are associated with more aggressive behavior in humans, and serotonergic supplementation is associated with greater agreeableness, cooperation, and affiliative behaviors in human and animal models^61,62^. In addition, MDMA’s dopaminergic activity might be implicated in the association between MDMA and lowered odds of social impairment, as evidence suggests that dopaminergic signaling promotes social approach behavior in the context of positive cues and plays a role in the sensitivity of rewards^62^.

#### Oxytocin release

Another potential mechanism by which MDMA/ecstasy is believed to increase sociability is through elevated oxytocin release, which may be mediated by the aforementioned changes in serotonergic neurotransmission^63^. Oxytocin is known to play an important role in facilitating social learning and social connection in mammals, including humans^64–66^. In a randomized controlled trial by Dumont et. al. aimed at assessing the relationship between MDMA/ecstasy administration and oxytocin release, MDMA/ecstasy was observed to increase both subjective prosocial feelings and oxytocin concentrations relative to placebo^66^. Moreover, increased prosocial feelings were more strongly correlated with elevated blood oxytocin levels than with blood MDMA/ecstasy levels, suggesting that the relationship between MDMA/ecstasy use and prosocial feelings might be distinctly mediated by oxytocin release. Given that oxytocin promotes trust and emotional connection, MDMA/ecstasy’s facilitation of its release might explain why MDMA/ecstasy users often feel more connected and prosocial^67,68^. Thus, increased social bonding via oxytocin might in part explain the association between MDMA/ecstasy and lowered odds of social impairments.

#### Decreased amygdala reactivity

MDMA/ecstasy may also contribute to increased sociability by reducing amygdala reactivity to social situations, an effect downstream of changes in serotonergic neurotransmission and oxytocin release^69,70^. The amygdala has been shown to be important in socioemotional processing, particularly for threat-related information^71^. In a 2009 fMRI study, Bedi et al., found that MDMA/ecstasy had the effect of diminishing amygdala reactivity toward angry facial expressions^72^. Suppression of the social threat fear response produced by the amygdala may be one explanation for the prosocial effects of MDMA/ecstasy. Furthermore, in the aforementioned study by Dumont et al. that found MDMA to simultaneously increase prosocial feelings and oxytocin levels, the authors also propose that decreased amygdala activity may mediate the association between oxytocin and prosocial feelings^66^. Specifically, these authors draw on prior evidence that indicates oxytocin may diminish the amygdala response to novel social encounters, ultimately increasing prosocial feelings and potentially decreasing social impairment^66,73^. Thus, decreased amygdala reactivity promoted by MDMA/ecstasy may also contribute to decreased fear of social interaction, ultimately lowering the odds of social impairment.

#### Increased social motivation

MDMA’s serotonergic and dopaminergic properties might also be related to experimentally observed increases in social motivation after ingestion of MDMA. Both self-report and behavioral measures suggest that MDMA promotes a desire to be with other individuals, and enhances intrinsic motivation to engage in social approach behavior^74^. Meanwhile, MDMA might also blunt the effects of perceived social rejection, an experience which often reduces motivation to engage socially^75^. Taken together, these factors might have the effect of promoting more effective socialization and greater social connection, thereby reducing the likelihood of social impairment in the long run.

#### Enhanced attention and reward response to positive social stimuli

Downstream of its promotion of dopaminergic neurotransmission and oxytocin release, MDMA has demonstrated the capacity to enhance attention and reward response to positive social stimuli, perhaps accounting for some of its observed prosocial effects. An increasingly wide body of research suggests that MDMA increases attention to positive facial expressions, heightens sensitivity to social reward cues as measured by ventral striatum activation, and reopens the window for social reward learning by means of neuroplastic changes in the nucleus accumbens^76–78^. It is possible that enhanced sensitivity to reward in the context of social interaction helps to promote both higher levels of social engagement, and the development of more effective, reward-conditioned social attunement.

#### Increased empathy

The empathy-promoting effects of MDMA/ecstasy, widely attested to in the research literature and perhaps downstream of changes in serotonergic neurotransmission, might also underlie our core finding that lifetime MDMA/ecstasy use was associated with lowered odds of impairments in social functioning^79^. In a 2017 pooled analysis, participants from 6 controlled studies were found to have enhanced emotional empathy as a result of MDMA/ecstasy use^80^. Moreover, in a 2014 trial that compared performance between participants given MDMA/ecstasy and participants given methylphenidate (Ritalin) on a variety of empathy-related tasks, only participants who received MDMA/ecstasy demonstrated emotional empathy for positive stimuli, as well as increased subjective empathogenic feelings^81^. Furthermore, in another placebo-controlled trial performed by Hysek et al., a wide battery of measures captured increases in both emotional empathy and prosocial orientation due to MDMA/ecstasy administration^27^. In particular, Hysek et al. found decreased competitive behavior and increased empathetic concern in male subjects^27^. Overall, this experimental evidence suggests that an increase in empathy induced by MDMA/ecstasy might promote prosociality.

### Potential explanations: classic psychedelics and lowered odds of social impairment

Despite the present study’s primary focus on protective associations between lifetime use of MDMA/ecstasy and impairments in social functioning, we also took an interest in whether lifetime use of classic psychedelics, including tryptamines such as psilocybin, phenethylamines like mescaline, and lysergamides like LSD, shared protective associations with social impairment as well. Notably, the one significant association between lifetime use of a classic psychedelic and lowered odds of social impairment involved mescaline, which, like MDMA/ecstasy, belongs to the class of phenethylamine compounds.

Two possible explanations can be offered to explain this distinctive pattern, in which mescaline, but no other classic psychedelics, confers lowered odds of social impairment. First, it is possible that because MDMA and mescaline belong to the same class of compounds (phenethylamines), the associations that mescaline shares with lowered odds of social impairment are downstream of the common pharmacological mechanisms of this class of substances. Accordingly, classic psychedelics in other compound classes such as psilocybin and LSD would therefore lack these associations with social impairment. Second, it is possible that the common, specific association of MDMA and mescaline with reduced odds of social impairment can be attributed to common pre-drug factors that unite MDMA and mescaline users, or at minimum, set mescaline users apart from users of other classic psychedelics. For example, similar to the above-mentioned link between higher extroversion and individuals who use MDMA at the pre-drug level^58^, it is possible that a similar pattern applies to its structural cousin, mescaline. Additionally, while mescaline was once a popular drug of choice among individuals who use classic psychedelics, use of mescaline is now relatively less common^82^—suggesting that mescaline users might be a distinct, self-selected group who might differ in pre-drug traits from those who use more common classic psychedelics like psilocybin and LSD. However, it is important to note that these two explanations are speculative and additional research is needed to better understand this pattern of results.

Next, we also plan to explore the potential mechanisms by which mescaline, in particular, and classic psychedelics, in general, might also confer lowered odds of impairments in social functioning. As little research has been conducted on mescaline specifically, we will review the literature on classic psychedelics with similar subjective effects, as research on these compounds can inform us about the potential effects of mescaline on social impairment.

#### Neurotransmitter-receptor-level effects

Mescaline acts primarily as an agonist of 5-HT2A receptors. While this mechanism stands apart from the serotonergic pharmacology of MDMA, it may play some role in the observed relationship between lifetime use of mescaline and lowered odds of social impairment, by way of serotonin’s documented role in empathy modulation, recognition of facial emotions, cooperation, and aggression^61,62^. Meanwhile, some evidence exists for mescaline’s role as a weak dopamine receptor agonist, but the nature of this link is tentative and uncertain^83^.

#### Decreased amygdala reactivity

Similar to MDMA/ecstasy, mescaline may promote sociability by occasioning reductions in amygdala reactivity, as well as shifts in emotional processing related to negative facial recognition. In a randomized controlled fMRI study by Mueller et al., lysergic acid diethylamide (LSD) was found to reduce reactivity of the left amygdala to fearful stimuli^84^. Importantly, previous studies have specifically implicated the left amygdala in the processing of negative facial expressions, relative to its counterpart to the right^85–88^. By reducing reactivity of the left amygdala to negative facial expressions, classic psychedelics, including mescaline, might help to reduce a felt sense of threat in the presence of others, thereby reducing impairments in social functioning.

#### Greater sense of interconnectedness

Additionally, mescaline, as well as other classic psychedelics, may promote prosociality and lower the odds of social impairment by promoting a felt sense of interconnectedness with other people^89^. In a follow-up of an open label study aimed at investigating the possible role of psilocybin-assisted therapy in promoting smoking cessation, participants also reported engaging in more prosocial behavior after treatment^90^. The authors theorized this effect to be mediated by self-reported increases in felt interconnectedness, which resulted in more prosocial action^90^. Furthermore, in an open label study that demonstrated psilocybin with psychological support to be an effective treatment for treatment-resistant depression^91^, participants also endorsed a greater sense of “connectedness” following this therapy as well^92^. By promoting a greater sense of interconnectedness, classic psychedelics, including mescaline, might help to ease social distress and ultimately lower the odds of impairments in social functioning.

### Limitations

Our findings must be interpreted in view of several limitations which have been named in other population-based survey studies^52,93,94^. First, we cannot infer causal relationships from our data, which are cross-sectional in nature. Longitudinal studies and randomized controlled trials are necessary to demonstrate a causal link between MDMA/ecstasy and mescaline use with lowered odds of impairments in social functioning.

Second, due to limitations in the NSDUH dataset, we were unable to control for all potential demographic confounds that may otherwise explain the link between MDMA/ecstasy use and lowered odds of impairments in social functioning. For example, the NSDUH does not survey individuals living in treatment centers, active duty military members, and incarcerated individuals. Furthermore, as previously mentioned in our Results, there are significant differences between those who have versus have not used MDMA and such differences may underlie our results, as individuals who have used MDMA tend to be male and Non-Hispanic White. Although we controlled for these demographic factors in our results, there may be other demographic factors related to individuals who use MDMA/ecstasy that we could not incorporate into our models. Overall, the absence of individuals from these unsampled populations, as well as other sources of demographic skew in the NSDUH sample, might limit the generalizability of our findings.

Third, pharmacological limitations must be taken into account, as determining the purity and authenticity of the MDMA/ecstasy ingested by NSDUH respondents is beyond the scope of our study. In a naturalistic context, doses of MDMA/ecstasy vary widely in their purity and in the amount of MDMA they contain^95,96^. The impure quality of naturalistic MDMA/ecstasy may weaken any pharmacological interpretations of our observed link between MDMA/ecstasy use and lowered odds of social impairments. It is notable, however, that in spite of this limitation, we still found evidence for a link between lifetime MDMA/ecstasy use and reduced impairments in social functioning, in line with the existing literature on the prosocial effects of MDMA/ecstasy. Randomized controlled trials using pure, laboratory-grade MDMA can address this limitation and help to establish a causal link between MDMA/ecstasy use and the alleviation of social impairment.

Fourth, it is possible harmful outcomes occurred due to MDMA/ecstasy or classic psychedelic use, causing or exacerbating social difficulties for some participants in this study. This idea is reinforced by our findings linking lifetime LSD use to increased odds of difficulty dealing with strangers and difficulty participating in social activities. Both MDMA/ecstasy and classic psychedelics have been associated with adverse outcomes in some instances. Although the evidence remains equivocal, MDMA/ecstasy use has been linked to neurotoxicity and lasting cognitive impairments with chronic use^97–99^. Additionally, MDMA/ecstasy can acutely cause adverse reactions such as anxiety and mood disturbance as well^100,101^.

Classic psychedelics have also been linked to adverse reactions upon acute administration—commonly referred to as “bad trips”—in which individuals experience panic, paranoia, extreme fear and distress, and anxiety during a psychedelic experience^102^. Furthermore, classic psychedelics have been tentatively linked to increased risk of psychosis^102–104^, although much of the evidence that supports this association is anecdotal and historical. These potential harms might also explain the lack of associations that we found in this study for psilocybin and peyote and social impairment. Although we previously described potential drivers by which classic psychedelics may alleviate social impairment, such drivers may be counterbalanced by these potential harms at the population level. Relatedly, there may be large sub-groups in the sample who are particularly vulnerable to these harms. Analyzing these groups alongside the broader sample, which includes individuals for whom classic psychedelics have a neutral or salutary effect, may have subsequently led to our null findings for these compounds. Overall, future investigations should examine the conditions under which MDMA/ecstasy and classic psychedelics can potentially promote harmful outcomes and contribute to impairments in social functioning.

Fifth, multicollinearity is a possible limitation to our study. Many of the substance use and demographic variables in our analyses likely share robust levels of correlation, and studies utilizing a virtually identical analytical structure have identified multicollinearity within these models^48^. The impact of multicollinearity on our results is largely mitigated by our large sample size, however. Multicollinearity serves to inflate the standard errors of one’s models and make it less likely for significance tests to reach significance, a fact that does not pose an issue for samples large enough to buffer against such inflation^105^. However, future studies can address this limitation by using analytical approaches that are designed to handle multicollinearity, such as ridge regression.

Sixth, this study design did not allow us to establish frequency of substance use or temporal precedence between MDMA/ecstasy and classic psychedelic use, on one hand, and decreased social difficulties related to mental health, on the other. However, psychedelics have demonstrated that they can promote salutary outcomes with few uses in therapeutic contexts^20,106^; thus it remains plausible that infrequent use may be associated with salutary outcomes in this study. Furthermore because MDMA/ecstasy use was assessed over a lifetime, it is likely that much of the MDMA/ecstasy use occurred prior to the onset of social difficulties as well. Future longitudinal studies and cross sectional studies featuring non-overlapping time horizons and more granular measures of substance use can more thoroughly address this limitation.

## Conclusion

The aim of this investigation was to assess whether associations existed between MDMA/ecstasy and classic psychedelic use (psilocybin, LSD, peyote, mescaline) and impairments in social functioning. Overall, we found lifetime MDMA/ecstasy use to be associated with lowered odds of the majority of our social impairment outcomes and lifetime mescaline use to confer lowered odds of difficulty dealing with strangers. LSD was associated with increased odds of two social impairment outcomes as well. Although this study cannot be used to determine a causal relationship between use of these substances and changes in social difficulties, this study can pave the way for future experimental studies that assess whether these substances can support social functioning, as well as others that allow for better understanding of potential risks related to these compounds as well. Overall, this study represents incremental progress in better supporting individuals experiencing social difficulties related to mental health disorders.

## Data Availability

The data for this project are publicly available at the Substance Abuse & Mental Health Data Archive (SAMHDA) at the following web address: https://www.datafiles.samhsa.gov/.

## References

[1] Sasamoto A, Miyata J, Hirao K (2011). Social impairment in schizophrenia revealed by Autism-Spectrum Quotient correlated with gray matter reduction. Soc. Neurosci.

[2] Constantino JN (2011). The quantitative nature of autistic social impairment. Pediatr. Res..

[3] Armijo J (2017). Social impairment and mental health. Ann. Behav. Sci..

[4] Rymaszewska J, Jarosz-Nowak J, Kiejna A, Kallert T, Schützwohl M, Priebe S, Wright D, Nawka P, Raboch J (2007). Social disability in different mental disorders. Eur. Psychiatry.

[5] Saris IMJ, Aghajani M, van der Werff SJA, van der Wee NJA, Penninx BWJH (2017). Social functioning in patients with depressive and anxiety disorders. Acta Psychiatr. Scand..

[6] Wang J, Mann F, Lloyd-Evans B, Ma R, Johnson S (2018). Associations between loneliness and perceived social support and outcomes of mental health problems: A systematic review. BMC Psychiatry.

[7] Wittchen H-U (2002). Generalized anxiety disorder: Prevalence, burden, and cost to society. Depress Anxiety.

[8] Bruce TJ, Saeed SA (1999). Social anxiety disorder: A common, underrecognized mental disorder. Am. Fam. Physician.

[9] Blanco C, Bragdon LB, Schneier FR, Liebowitz MR (2013). The evidence-based pharmacotherapy of social anxiety disorder. Int. J. Neuropsychopharmacol..

[10] Maddox BB, Miyazaki Y, White SW (2017). Long-term effects of CBT on social impairment in adolescents with ASD. J. Autism Dev. Disord..

[11] Canton J, Scott KM, Glue P (2012). Optimal treatment of social phobia: Systematic review and meta-analysis. Neuropsychiatr. Dis. Treat..

[12] Yui K, Koshiba M, Nakamura S, Kobayashi Y (2012). Effects of large doses of arachidonic acid added to docosahexaenoic acid on social impairment in individuals with autism spectrum disorders: A double-blind, placebo-controlled, randomized trial. J. Clin. Psychopharmacol..

[13] Bandelow B (2020). Current and novel psychopharmacological drugs for anxiety disorders. Adv. Exp. Med. Biol..

[14] Cottraux J (2005). Recent developments in research and treatment for social phobia (social anxiety disorder). Curr. Opin. Psychiatry.

[15] Sartori SB, Singewald N (2019). Novel pharmacological targets in drug development for the treatment of anxiety and anxiety-related disorders. Pharmacol. Ther..

[16] Schenberg EE (2018). Psychedelic-assisted psychotherapy: A paradigm shift in psychiatric research and development. Front. Pharmacol..

[17] Kalant H (2001). The pharmacology and toxicology of “ecstasy” (MDMA) and related drugs. CMAJ Can. Med. Assoc. J..

[18] Nichols DE (2016). Psychedelics. Pharmacol. Rev..

[19] Sessa B, Higbed L, Nutt D (2019). A review of 3,4-methylenedioxymethamphetamine (MDMA)-assisted psychotherapy. Front. Psychiatry.

[20] Mitchell JM, Bogenschutz M, Lilienstein A (2021). MDMA-assisted therapy for severe PTSD: A randomized, double-blind, placebo-controlled phase 3 study. Nat. Med..

[21] Mithoefer MC, Wagner MT, Mithoefer AT, Jerome L, Martin SF, Yazar-Klosinski B, Michel Y, Brewerton TD, Doblin R (2013). Durability of improvement in post-traumatic stress disorder symptoms and absence of harmful effects or drug dependency after 3,4-methylenedioxymethamphetamine-assisted psychotherapy: A prospective long-term follow-up study. J. Psychopharmacol..

[22] Mithoefer MC, Feduccia AA, Jerome L, Mithoefer A, Wagner M, Walsh Z, Hamilton S, Yazar-Klosinski B, Emerson A, Doblin R (2019). MDMA-assisted psychotherapy for treatment of PTSD: Study design and rationale for phase 3 trials based on pooled analysis of six phase 2 randomized controlled trials. Psychopharmacology.

[23] Ot’alora GM, Grigsby J, Poulter B (2018). 3,4-Methylenedioxymethamphetamine-assisted psychotherapy for treatment of chronic posttraumatic stress disorder: A randomized phase 2 controlled trial. J. Psychopharmacol..

[24] Mithoefer MC, Wagner MT, Mithoefer AT, Jerome L, Doblin R (2011). The safety and efficacy of ±3,4-methylenedioxymethamphetamine-assisted psychotherapy in subjects with chronic, treatment-resistant posttraumatic stress disorder: The first randomized controlled pilot study. J. Psychopharmacol..

[25] Regan A, Margolis S, de Wit H, Lyubomirsky S (2021). Does ±3,4-methylenedioxymethamphetamine (ecstasy) induce subjective feelings of social connection in humans? A multilevel meta-analysis. PLoS ONE.

[26] Kamilar-Britt P, Bedi G (2015). The prosocial effects of 3,4-methylenedioxymethamphetamine (MDMA): Controlled studies in humans and laboratory animals. Neurosci. Biobehav. Rev..

[27] Hysek CM, Schmid Y, Simmler LD, Domes G, Heinrichs M, Eisenegger C, Preller KH, Quednow BB, Liechti ME (2014). MDMA enhances emotional empathy and prosocial behavior. Soc. Cogn. Affect. Neurosci..

[28] Bedi G, Hyman D, de Wit H (2010). Is ecstasy an “empathogen”? Effects of ±3,4-methylenedioxymethamphetamine on prosocial feelings and identification of emotional states in others. Biol. Psychiatry.

[29] Heifets BD, Malenka RC (2016). MDMA as a probe and treatment for social behaviors. Cell.

[30] Danforth AL, Grob CS, Struble C, Feduccia AA, Walker N, Jerome L, Yazar-Klosinski B, Emerson A (2018). Reduction in social anxiety after MDMA-assisted psychotherapy with autistic adults: A randomized, double-blind, placebo-controlled pilot study. Psychopharmacology.

[31] Griffiths RR, Richards WA, McCann U, Jesse R (2006). Psilocybin can occasion mystical-type experiences having substantial and sustained personal meaning and spiritual significance. Psychopharmacology.

[32] James E, Robertshaw TL, Hoskins M, Sessa B (2020). Psilocybin occasioned mystical-type experiences. Hum. Psychopharmacol. Clin. Exp..

[33] Johnson MW, Garcia-Romeu A, Cosimano MP, Griffiths RR (2014). Pilot study of the 5-HT2AR agonist psilocybin in the treatment of tobacco addiction. J. Psychopharmacol..

[34] Bogenschutz MP, Forcehimes AA, Pommy JA, Wilcox CE, Barbosa P, Strassman RJ (2015). Psilocybin-assisted treatment for alcohol dependence: A proof-of-concept study. J. Psychopharmacol..

[35] Carhart-Harris R, Giribaldi B, Watts R, Baker-Jones M, Murphy-Beiner A, Murphy R, Martell J, Blemings A, Erritzoe D, Nutt DJ (2021). Trial of psilocybin versus escitalopram for depression. N. Engl. J. Med..

[36] Davis AK, Barrett FS, May DG, Cosimano MP, Sepeda ND, Johnson MW, Finan PH, Griffiths RR (2020). Effects of psilocybin-assisted therapy on major depressive disorder: A randomized clinical trial. JAMA Psychiat..

[37] Griffiths RR, Johnson MW, Carducci MA, Umbricht A, Richards WA, Richards BD, Cosimano MP, Klinedinst MA (2016). Psilocybin produces substantial and sustained decreases in depression and anxiety in patients with life-threatening cancer: A randomized double-blind trial. J. Psychopharmacol..

[38] Dolder PC, Schmid Y, Müller F, Borgwardt S, Liechti ME (2016). LSD acutely impairs fear recognition and enhances emotional empathy and sociality. Neuropsychopharmacology.

[39] Griffiths RR, Johnson MW, Richards WA, Richards BD, Jesse R, MacLean KA, Barrett FS, Cosimano MP, Klinedinst MA (2018). Psilocybin-occasioned mystical-type experience in combination with meditation and other spiritual practices produces enduring positive changes in psychological functioning and in trait measures of prosocial attitudes and behaviors. J. Psychopharmacol..

[40] Preller KH, Schilbach L, Pokorny T, Flemming J, Seifritz E, Vollenweider FX (2018). Role of the 5-HT2A receptor in self- and other-initiated social interaction in lysergic acid diethylamide-induced states: A pharmacological fMRI study. J. Neurosci..

[41] Preller KH, Vollenweider FX (2019). Modulation of social cognition via hallucinogens and “entactogens”. Front. Psychiatry.

[42] Markopoulos A, Inserra A, De Gregorio D, Gobbi G (2022). Evaluating the potential use of serotonergic psychedelics in autism spectrum disorder. Front. Pharmacol..

[43] United States Department of Health and Human Services. Substance Abuse and Mental Health Services Administration. Center for Behavioral Health Statistics and Quality. *National Survey on Drug Use and Health* (2015).

[44] United States Department of Health and Human Services. Substance Abuse and Mental Health Services Administration. Center for Behavioral Health Statistics and Quality. *National Survey on Drug Use and Health* (2016).

[45] United States Department of Health and Human Services. Substance Abuse and Mental Health Services Administration. Center for Behavioral Health Statistics and Quality. *National Survey on Drug Use and Health* (2017).

[46] United States Department of Health and Human Services. Substance Abuse and Mental Health Services Administration. Center for Behavioral Health Statistics and Quality. *National Survey on Drug Use and Health* (2018).

[47] United States Department of Health and Human Services. Substance Abuse and Mental Health Services Administration. Center for Behavioral Health Statistics and Quality. *National Survey on Drug Use and Health* (2019).

[48] Jones G, Lipson J, Nock MK (2022). Associations between classic psychedelics and nicotine dependence in a nationally representative sample. Sci. Rep..

[49] Jones G, Ricard JA, Lipson J, Nock MK (2022). Associations between classic psychedelics and opioid use disorder in a nationally-representative U.S. adult sample. Sci. Rep..

[50] Jones GM, Nock MK (2022). Lifetime use of MDMA/ecstasy and psilocybin is associated with reduced odds of major depressive episodes. J. Psychopharmacol..

[51] Jones GM, Nock MK (2022). Psilocybin use is associated with lowered odds of crime arrests in US adults: A replication and extension. J. Psychopharmacol..

[52] Jones GM, Nock MK (2022). MDMA/ecstasy use and psilocybin use are associated with lowered odds of psychological distress and suicidal thoughts in a sample of US adults. J. Psychopharmacol..

[53] Jones GM, Nock MK (2022). Exploring protective associations between the use of classic psychedelics and cocaine use disorder: A population-based survey study. Sci. Rep..

[54] Lumley T (2020). survey: Analysis of complex survey samples. J. Stat. Softw..

[55] Sjoberg DD, Whiting K, Curry M, Lavery JA, Larmarange J (2021). Reproducible summary tables with the gtsummary package. R J..

[56] Jones G, Arias D, Nock M (2022). Associations between MDMA/ecstasy, classic psychedelics, and suicidal thoughts and behaviors in a sample of U.S. adolescents. Sci. Rep..

[57] Jones G, Ricard JA, Hendricks P, Simonsson O (2022). Associations between MDMA/ecstasy use and physical health in a U.S. population-based survey sample. J. Psychopharmacol..

[58] ter Bogt TFM, Engels RCME, Dubas JS (2006). Party people: Personality and MDMA use of house party visitors. Addict. Behav..

[59] Nour MM, Evans L, Carhart-Harris RL (2017). Psychedelics, personality and political perspectives. J. Psychoact. Drugs.

[60] Hildebrandt A, Kly A, Reuter M, Sommer W, Wilhelm O (2016). Face and emotion expression processing and the serotonin transporter polymorphism 5-HTTLPR/rs22531. Genes Brain Behav..

[61] Kiser D, Steemers B, Branchi I, Homberg J (2012). The reciprocal interaction between serotonin and social behaviour. Neurosci. Biobehav. Rev..

[62] Enter D, Colzato LS, Roelofs K (2012). Dopamine transporter polymorphisms affect social approach-avoidance tendencies. Genes Brain Behav..

[63] Crockett M, Clark L, Hauser M, Robbins T (2010). Serotonin selectively influences moral judgment and behavior through effects on harm aversion. PNAS.

[64] Campbell A (2008). Attachment, aggression and affiliation: The role of oxytocin in female social behavior. Biol. Psychol..

[65] Hurlemann R, Patin A, Onur OA (2010). Oxytocin enhances amygdala-dependent, socially reinforced learning and emotional empathy in humans. J. Neurosci..

[66] Dumont GJH, Sweep FCGJ, van der Steen R, Hermsen R, Donders ART, Touw DJ, van Gerven JMA, Buitelaar JK, Verkes RJ (2009). Increased oxytocin concentrations and prosocial feelings in humans after ecstasy (3,4-methylenedioxymethamphetamine) administration. Soc. Neurosci..

[67] Kosfeld M, Heinrichs M, Zak PJ, Fischbacher U, Fehr E (2005). Oxytocin increases trust in humans. Nature.

[68] MacDonald K, MacDonald TM (2010). The peptide that binds: A systematic review of oxytocin and its prosocial effects in humans. Harv. Rev. Psychiatry.

[69] Bocchio M, McHugh S, Bannerman D, Sharp T, Capogna M (2016). Serotonin, amygdala and fear: Assembling the puzzle. Front. Neural Circuits.

[70] Sobota R, Mihara T, Forrest A, Featherstone R, Siegel S (2015). Oxytocin reduces amygdala activity, increases social interactions and reduces anxiety-like behavior irrespective of NMDAR antagonism. Behav. Neurosci..

[71] Zald DH (2003). The human amygdala and the emotional evaluation of sensory stimuli. Brain Res. Rev..

[72] Bedi G, Phan KL, Angstadt M, de Wit H (2009). Effects of MDMA on sociability and neural response to social threat and social reward. Psychopharmacology.

[73] Baumgartner T, Heinrichs M, Vonlanthen A, Fischbacher U, Fehr E (2008). Oxytocin shapes the neural circuitry of trust and trust adaptation in humans. Neuron.

[74] Luoma J, Lear K (2021). MDMA-assisted therapy as a means to alter affective, cognitive, behavioral, and neurological systems underlying social dysfunction in social anxiety disorder. Front. Psychiatry.

[75] Frye C, Wardle M, Norman G, de Wit H (2014). MDMA decreases the effects of simulated social rejection. Pharmacol. Biochem. Behav..

[76] Bershad A, Mayo L, Van Hedger K, McGlone F, Walker S, de Wit H (2019). Effects of MDMA on attention to positive social cues and pleasantness of affective touch. Neuropsychopharmacology.

[77] Wardle MC, Kirkpatrick MG, de Wit H (2014). ‘Ecstasy’ as a social drug: MDMA preferentially affects responses to emotional stimuli with social content. Soc. Cogn. Affect. Neurosci..

[78] Nardou R, Lewis E, Rothaas R, Xu R, Yang A, Boyden E, Dölen G (2019). Oxytocin-dependent reopening of a social reward learning critical period with MDMA. Nature.

[79] Gong P, Liu J, Blue P, Li S, Zhou X (2015). Serotonin receptor gene (HTR2A) T102C polymorphism modulates individuals’ perspective taking ability and autistic-like traits. Front. Hum. Neurosci..

[80] Kuypers KP, Dolder PC, Ramaekers JG, Liechti ME (2017). Multifaceted empathy of healthy volunteers after single doses of MDMA: A pooled sample of placebo-controlled studies. J. Psychopharmacol..

[81] Schmid Y, Hysek CM, Simmler LD, Crockett MJ, Quednow BB, Liechti ME (2014). Differential effects of MDMA and methylphenidate on social cognition. J. Psychopharmacol..

[82] Uthaug MV, Davis AK, Haas TF, Davis D, Dolan SB, Lancelotta R, Timmermann C, Ramaekers JG (2022). The epidemiology of mescaline use: Pattern of use, motivations for consumption, and perceived consequences, benefits, and acute and enduring subjective effects. J. Psychopharmacol..

[83] Kolaczynska K, Luethi D, Trachsel D, Hoener M, Liechti M (2022). Receptor interaction profiles of 4-alkoxy-3,5-dimethoxy-phenethylamines (mescaline derivatives) and related amphetamines. Front. Pharmacol..

[84] Mueller F, Lenz C, Dolder PC, Harder S, Schmid Y, Lang UE, Liechti ME, Borgwardt S (2017). Acute effects of LSD on amygdala activity during processing of fearful stimuli in healthy subjects. Transl. Psychiatry.

[85] Blair RJR, Morris JS, Frith CD, Perrett DI, Dolan RJ (1999). Dissociable neural responses to facial expressions of sadness and anger. Brain.

[86] Hardee JE, Thompson JC, Puce A (2008). The left amygdala knows fear: Laterality in the amygdala response to fearful eyes. Soc. Cogn. Affect. Neurosci..

[87] Phillips ML, Medford N, Young AW, Williams L, Williams SCR, Bullmore ET, Gray JA, Brammer MJ (2001). Time courses of left and right amygdalar responses to fearful facial expressions. Hum. Brain Mapp..

[88] Tuulari JJ, Kataja E-L, Leppänen JM (2020). Newborn left amygdala volume associates with attention disengagement from fearful faces at eight months. Dev. Cogn. Neurosci..

[89] Carhart-Harris RL, Erritzoe D, Haijen E, Kaelen M, Watts R (2018). Psychedelics and connectedness. Psychopharmacology.

[90] Noorani T, Garcia-Romeu A, Swift TC, Griffiths RR, Johnson MW (2018). Psychedelic therapy for smoking cessation: Qualitative analysis of participant accounts. J. Psychopharmacol..

[91] Carhart-Harris RL, Bolstridge M, Rucker J (2016). Psilocybin with psychological support for treatment-resistant depression: An open-label feasibility study. Lancet Psychiatry.

[92] Watts R, Day C, Krzanowski J, Nutt D, Carhart-Harris R (2017). Patients’ accounts of increased “connectedness” and “acceptance” after psilocybin for treatment-resistant depression. J. Humanist Psychol..

[93] Hendricks PS, Thorne CB, Clark CB, Coombs DW, Johnson MW (2015). Classic psychedelic use is associated with reduced psychological distress and suicidality in the United States adult population. J. Psychopharmacol..

[94] Sexton JD, Nichols CD, Hendricks PS (2020). Population survey data informing the therapeutic potential of classic and novel phenethylamine, tryptamine, and lysergamide psychedelics. Front. Psychiatry.

[95] Jalali A, Hatamie A, Saferpour T, Khajeamiri A, Safa T, Buazar F (2016). Impact of pharmaceutical impurities in ecstasy tablets: Gas chromatography-mass spectrometry study. Iran. J. Pharm. Res. IJPR.

[96] Saleemi S, Pennybaker SJ, Wooldridge M, Johnson MW (2017). Who is “Molly”? MDMA adulterants by product name and the impact of harm-reduction services at raves. J. Psychopharmacol..

[97] Gouzoulis-Mayfrank E, Daumann J (2006). Neurotoxicity of methylenedioxyamphetamines (MDMA; ecstasy) in humans: How strong is the evidence for persistent brain damage?. Addiction.

[98] Morgan MJ (2000). Ecstasy (MDMA): A review of its possible persistent psychological effects. Psychopharmacology.

[99] Costa G, Gołembiowska K (2022). Neurotoxicity of MDMA: Main effects and mechanisms. Exp. Neurol..

[100] Sarkar S, Schmued L (2010). Neurotoxicity of ecstasy (MDMA): An overview. Curr. Pharm. Biotechnol..

[101] Meyer JS (2013). 3,4-methylenedioxymethamphetamine (MDMA): Current perspectives. Subst. Abuse Rehabil..

[102] Johnson MW, Richards WA, Griffiths RR (2008). Human hallucinogen research: Guidelines for safety. J. Psychopharmacol..

[103] Johnson MW, Griffiths RR, Hendricks PS, Henningfield JE (2018). The abuse potential of medical psilocybin according to the 8 factors of the Controlled substances act. Neuropharmacology.

[104] Paparelli A, Di Forti M, Morrison P, Murray R (2011). Drug-induced psychosis: How to avoid star gazing in schizophrenia research by looking at more obvious sources of light. Front. Behav. Neurosci..

[105] Gujarati DN (2003). Basic Econometrics.

[106] Grob CS, Danforth AL, Chopra GS, Hagerty M, McKay CR, Halberstadt AL, Greer GR (2011). Pilot study of psilocybin treatment for anxiety in patients with advanced-stage cancer. Arch. Gen. Psychiatry.

